# Revisiting the *Plasmodium falciparum *RIFIN family: from comparative genomics to 3D-model prediction

**DOI:** 10.1186/1471-2164-10-445

**Published:** 2009-09-21

**Authors:** Emanuele Bultrini, Kevin Brick, Srayanta Mukherjee, Yang Zhang, Francesco Silvestrini, Pietro Alano, Elisabetta Pizzi

**Affiliations:** 1 Dipartimento di Malattie Infettive, Parassitarie ed Immunomediate, Istituto Superiore di Sanità, Viale Regina Elena, 299, 00161 Roma, Italy; 2 University of Kansas, Department of Molecular Biosciences Center for Bioinformatics 2030 Becker D0 Lawrence, KS 66047-1620, USA

## Abstract

**Background:**

Subtelomeric *RIFIN *genes constitute the most abundant multigene family in *Plasmodium falciparum*. *RIFIN *products are targets for the human immune response and contribute to the antigenic variability of the parasite. They are transmembrane proteins grouped into two sub-families (RIF_A and RIF_B). Although recent data show that RIF_A and RIF_B have different sub-cellular localisations and possibly different functions, the same structural organisation has been proposed for members of the two sub-families. Despite recent advances, our knowledge of the regulation of *RIFIN *gene expression is still poor and the biological role of the protein products remain obscure.

**Results:**

Comparative studies on *RIFINs *in three clones of *P. falciparum *(3D7, HB3 and Dd2) by Multidimensional scaling (MDS) showed that gene sequences evolve differently in the 5'upstream, coding, and 3'downstream regions, and suggested a possible role of highly conserved 3' downstream sequences. Despite the expected polymorphism, we found that the overall structure of *RIFIN *repertoires is conserved among clones suggesting a balance between genetic drift and homogenisation mechanisms which guarantees emergence of novel variants but preserves the functionality of genes. Protein sequences from a *bona fide *set of 3D7 RIFINs were submitted to predictors of secondary structure elements. In contrast with the previously proposed structural organisation, no signal peptide and only one transmembrane helix were predicted for the majority of RIF_As. Finally, we developed a strategy to obtain a reliable 3D-model for RIF_As. We generated 265 possible structures from 53 non-redundant sequences, from which clustering and quality assessments selected two models as the most representative for putative RIFIN protein structures.

**Conclusion:**

First, comparative analyses of *RIFIN *repertoires in different clones of *P. falciparum *provide insights on evolutionary mechanisms shaping the multigene family. Secondly, we found that members of the two sub-families RIF_As and RIF_Bs have different structural organization in accordance with recent experimental results. Finally, representative models for RIF_As have an "Armadillo-like" fold which is known to promote protein-protein interactions in diverse contexts.

## Background

Malaria is one of the most important infectious diseases in tropical and subtropical areas of the world, causing millions of deaths in developing countries every year [1]. The most severe agent of human malaria is *Plasmodium falciparum*, whose virulence is largely due to the capability of infected erythrocytes to adhere to host cell receptors and avoid splenic clearance [2]. Once the parasite enters a host erythrocyte, the cell undergoes a series of important modifications, one of which is the insertion of parasite proteins into the erythrocyte membrane to form knob-like structures. Knobs play a fundamental role in the pathogenesis of the disease. These structures are responsible for sequestration within the micro-vasculature of vital organs such as the brain (causing the cerebral form of malaria) and for the rosetting process that are related to severe forms of malaria [3].

The most studied knob-associated protein is the erythrocyte membrane protein-1 (PfEMP1), which is directly involved in the interactions between infected erythrocytes and host cell receptors. *Var *genes encoding PfEMP1 proteins are present in 50-60 copies in the parasite genome and are predominantly located in the subtelomeric regions of all 14 chromosomes [4,5]. PfEMP1 proteins are the main component of the variant surface antigens (VSA) that are responsible for the antigenic variation of the parasites. The expression of novel variants of these proteins at the surface of infected erythrocytes allows the parasite to evade the immune system and hence to proliferate in the human host [6]. In addition, it was demonstrated that their subtelomeric localisation promotes the continuous generation of new repertoires of proteins with new antigenic and adhesive properties [7,8]. In fact, frequent recombinations followed by duplication and gene conversion events lead to a reshuffling of subtelomeric regions which appear as a mosaic of sequences.

Linked to *var *genes at subtelomeres are members of the multigene family *RIFIN/STEVOR *[9,10]. Although sequence similarity reveals a common ancestor for *STEVORs *and *RIFINs*, recent studies of *STEVOR *transcription and expression show that they are distinct from the *RIFIN *multigene family, suggesting a different role for members of the two families [11].

*RIFIN *genes form the most abundant multigene family in *P. falciparum *with about 160 copies in the 3D7 genome. It has been shown that *RIFINs *are targets for the human immune response and are part of the VSA contributing to the antigenic variability of the parasite [12,13]. *RIFINs *encode transmembrane proteins with a predicted size of about 30-45 kid. Proteins can be grouped into two sub-families (RIF_A and RIF_B) as recognised on the basis of preliminary genome sequencing data by Pizzi et al. [14] and recently confirmed by Joannin et al. on the complete repertoire of members [15]. Apart from a short insertion (25 aa long) present only in RIF_A, a similar overall architectural organization has been proposed for members of the two sub-families: a signal peptide at the N-terminus, a PEXEL/VTS motif which is necessary to target proteins outside the parasite cell [16], and two transmembrane domains. However, it has been recently shown that RIF_As and RIF_Bs have different sub-cellular localisations and possibly different functions. RIF_As are localised outside the parasite cell, while RIF_Bs remain confined within the parasitophorous vacuole (PV). Furthermore, RIFIN variants have different developmental expression in merozoites where members of the two sub-families remain within the parasite cell although exhibiting different localisation patterns [17]. Evolutionary studies support these recent experimental data. McInerney and colleagues [18] showed that members of the *RIFIN *family are subject to different selective pressures, and suggested that this is due to their different exposure to the host immune system.

In this work, we present a study on the entire repertoire of *RIFINs *in genomes from three *P. falciparum *clones: 3D7, HB3 and Dd2. Each *RIFIN *gene in the three genomes was considered as an arrangement of three modules: 5' upstream sequences (1 Kb; *5ups*), coding sequences (*cds*) and 3' downstream sequences (1 Kb; *3dwn*). In order to carry out comparisons in each class of sequences, we used a Multidimensional Scaling (MDS) statistical method, which permits the study of relationships between sequences without any *a priori *assumptions as in the case of molecular evolutionary approaches. We found that *RIFIN *sequences (*5ups*, *cds *and *3dwn*) show a very similar cluster organisation in the three examined clones. We also proposed a classification of *RIFIN *genes based on the arrangement of the diverse type of module sequences and found that, despite the high expected variability, 3D7, HB3 and Dd2 share very similar repertoires of *RIFINs*. In the second part of the work, we submitted 159 *bona fide *RIFIN protein sequences from 3D7 to signal peptide and transmembrane predictors. Notably, we found that while RIF_Bs have a signal peptide, a PEXEL/VTS motif and two transmembrane domains, the great majority of RIF_As do not have a signal peptide and contain only one transmembrane domain was predicted at the C-terminus. These results therefore indicate that the RIF_A and RIF_B proteins do not share the currently accepted common domain organisation proposed for this multigene family. On the other hand these results support the recent experimental data on different sub-cellular localisations and possible different functions of RIF_As and RIF_Bs [17].

Finally, we constructed a 3D-model for RIF_As with the aim of gaining insights into the domains potentially involved of RIFINs in host-parasite interactions. Taking advantage of the large number of sequences we developed a strategy based on *ab initio *structure prediction by the I-TASSER algorithm [19,20]. 265 possible models were predicted for a subset of 53 non-redundant sequences and then clustered according to their fold similarity by TM-ALIGN [21]. We found that the two most representative models obtained in this analysis resemble the "Armadillo-like" fold.

## Results

### Multidimensional scaling (MDS) of *RIFIN *nucleotide sequences

In the 3D7 genome (PlasmoDB v5.4), 218 predicted gene products are annotated as members of the *RIFIN/STEVOR *family (PFAM: PF02009) of which 180 are *RIFINs*. In order to select *bona fide *RIFINs, we discarded products of truncated variants and pseudogenes and considered only those from two-exon genes with good alignments to the Pfam family profile (evalue < 1e^-10^). In this way we obtained a list of 159 protein sequences (see Additional file 1). In order to carry out a comparative analysis between RIFIN repertoires in different *P. falciparum *clones, 131 RIFIN/STEVOR proteins for HB3 and 156 for Dd2 were downloaded from the Broad Institute . Since for both genomes the annotation status is preliminary, we decided to use 3D7 *bona fide *RIFINs as a reference.

As standard matrices are not suited to compare proteins with biased amino acid compositions [22], recently novel series of adjusted matrices have been constructed specifically for *Plasmodium* proteins [23-25]. We used the CCF53 substitution matrix [24] to align the protein sequences from different clones using BLAST (3D7 *vs*. Dd2 and 3D7 *vs*. HB3). Only proteins with a percent identity (%ID) higher than 30% and an e-value lower than 1e^-10 ^were retained, which allowed us to discard uncertain RIFIN and STEVOR members. 105 and 108 *RIFIN *genes for HB3 and Dd2 respectively were thus identified for analysis (see Additional file 1).

Corresponding *RIFIN *gene sequences from three clones were considered and subdivided in three regions for analysis: 1 kb upstream the ATG codon (*ups*), the coding region (*cds*) and 1 kb downstream the stop codon (*dwn*). We first performed Needleman-Wunsch alignments [26] for all possible pairwise comparisons of *RIFIN *sequences from 3D7, HB3 and Dd2 and derived the percentage of identity between each pair of sequences. For each group of sequences (*ups, cds *and *dwn*), distance matrices were then constructed where d = 100-%ID. We used these matrices as input for MDS. MDS is a statistical method which allows mapping of sequences as points on a plane in which euclidean distances reflect those in the matrices (see methods for details). This permits the identification of possible clusters of sequences and hence the study of their relationships.

**Figure 1:**
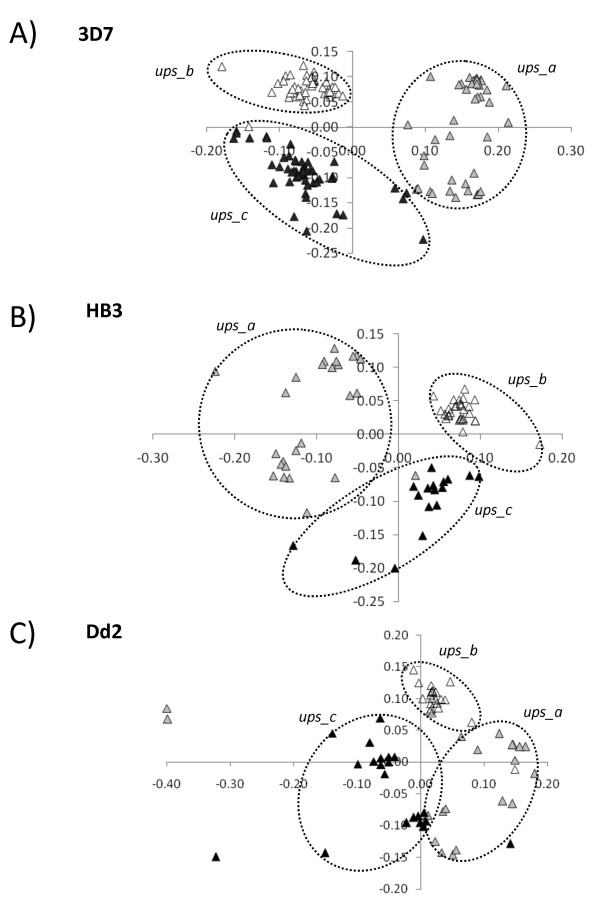
**Results of Multidimensional scaling (MDS) on 5' upstream sequences (1 kb) of 3D7 (panel A), HB3 (panel B) and Dd2 (panel C) RIFIN genes**. Colors are used to highlight members of the three clusters: *ups_a *(grey), *ups_b *(white), *ups_c *(black). In panels B and C dots are colored according the cluster classification proposed in 3D7; for example, empty triangles (*ups_b*) represent homologous sequences to *ups_b *sequences in 3D7.

MDS on 3D7, HB3 and Dd2 upstream regions revealed 11 sequences scattered on the plane, distant from the cluster containing the majority of sequences. Since the presence of outliers can interfere with the recognition of real clusters, we removed these sequences (see methods for details). When MDS was then repeated sequences appear to be organised into three groups and a k-means clustering (k = 3) was used to better define borders of clusters (see figure 1 panel A). Clusters named *ups_a*, *ups_b *and *ups_c *contain 43, 55 and 49 sequences respectively. Also in the case of the other two clones, we found several outliers (32 in HB3 and 46 in Dd2) which were removed before to repeat MDS. In figure 1, panels B and C, we report results for similar analysis of *RIFINs *in HB3 and Dd2. Despite the expected high variability, a very similar cluster organisation was found in all the three clones.

**Figure 2:**
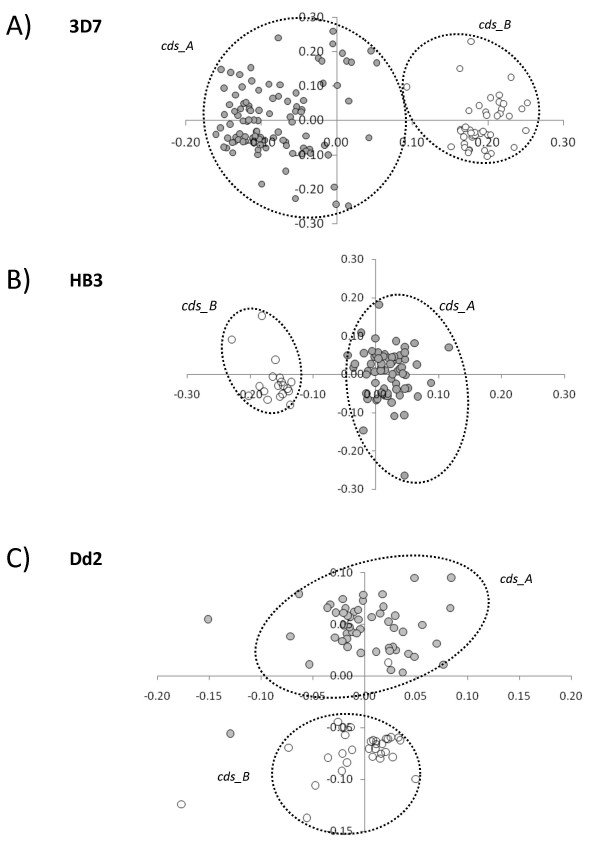
**Results of Multidimensional scaling (MDS) on coding sequences of 3D7 (panel A), HB3 (panel B) and Dd2 (panel C) RIFIN genes**. Colors are used to highlight members of the two clusters: *cds_A *(grey), *cds_B *(white).

Also in the case of coding sequences several outliers were discarded (12 sequences in 3D7, 29 in HB3 and 0 in Dd2). Results for 3D7 (figure 2 panel A) confirm that *RIFINs *are organised into two sub-families as previously described [14,15]. In accordance with classification proposed by Joannin et al. [15] we indicated the two groups of sequences *cds_A *and *cds_B*. *cds_A *correspond to 100 genes coding for RIF_A which are characterised by the presence of a specific insert in protein products, while *cds_B *are genes coding for 44 members of the sub-family RIF_B. The same organisation into two sub-families is maintained also in HB3 (panel B) and Dd2 (panel C), although more scattered distributions of sequences are observed (especially for *cds_A*).

**Figure 3:**
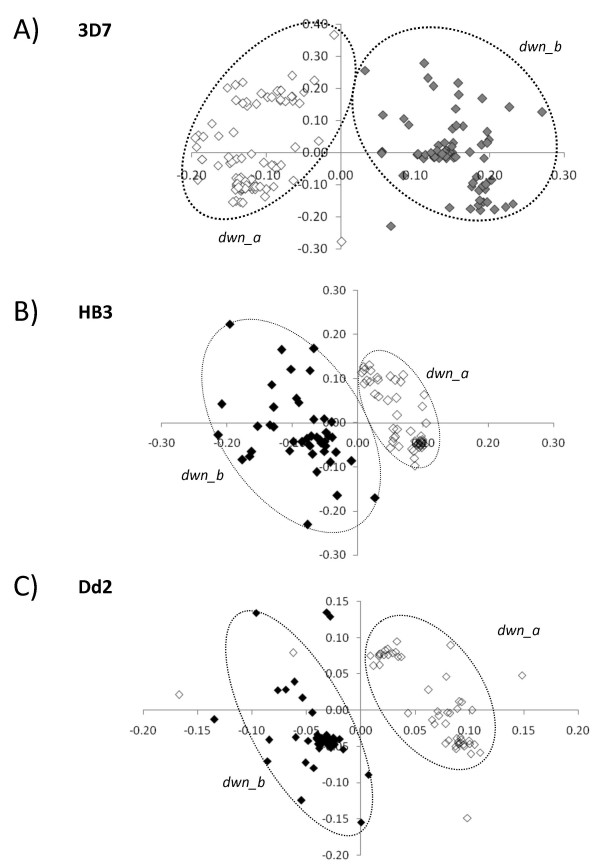
**Results of Multidimensional scaling (MDS) on 3' downstream sequences (1 kb) of 3D7 (panel A), HB3 (panel B) and Dd2 (panel C) RIFIN genes**. Colors are used to highlight members of the two clusters: *dwn_a *(grey), *dwn_b *(white). (The grey in fig.3A is different to that in fig3B and 3C)

In the case of 3' downstream sequences of 3D7 (see figure 3, panel A) we did not find any outliers, and the sequences appear clearly clustered into two groups (89 *dwn_a *and 70 *dwn_b*). In HB3 and Dd2 only few outliers were removed (5 sequences in HB3 and 12 Dd2) and MDS showed the same cluster organisation obtained in 3D7 (figure 3, panels B and C). This MDS analysis has established that *RIFIN *gene sequences maintain a very similar cluster organisation in all three of the examined genomes. In addition, differences in the number of outliers for *ups*, *cds *and *dwn *sequences revealed that these regions evolve differently. In particular, the few outliers in *dwn *sequences (0 in 3D7, 5 in HB3 and 12 in Dd2) show that these regions are highly conserved among members of the multigene family.

### *RIFIN *repertoires in 3D7, HB3 and Dd2

**Figure 4:**
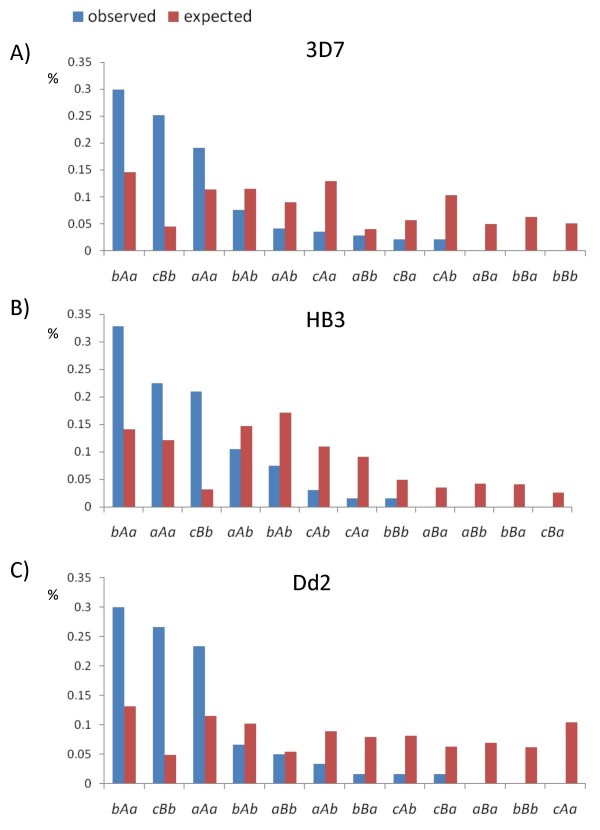
***RIFIN *genes were classified according to the arrangement of upstream (*ups*), coding (*cds*) and downstream (*dwn*) sequences**. Observed frequencies (blue bars) of gene arrangements are compared with those expected (red bars) on the basis of a probabilistic model.

MDS results described above allow a novel classification of *RIFIN *genes based on the arrangement of the diverse types of *ups*, *cds *and *dwn *modules. Under the hypothesis that modules might occur independently of each other, the probability for a given combination is the product of the three frequencies at which *ups, cds *and *dwn *occur in the genome, that is *f(ups-cds-dwn) *= *f(ups)·f(cds)·f(dwn)*. We calculated the frequencies at which each of the 12 possible module arrangements are expected in a genome and compared them with the observed ones (figure 4). We observed that *RIFIN *repertoires have highly similar gene distributions in 3D7, HB3 and Dd2. Furthermore, in the three genomes examined, these distributions strongly deviate from the random expectation (p < 10^-20^). In particular, the most frequent combinations are *bAa*, *cBb *and *aAa *which are significantly over-represented. The ratio between observed and expected frequencies of combinations *bAa *and *aAa *is higher than 1.5 in all the three genomes, and ranges from 5.6 to 9.4 for combination *cBb*.

Furthermore, accordingly to our novel classification, we examined the genomic localization of *RIFINs *in 3D7 [27]. We found that the most frequent *RIFIN *genes (*bAa, cBb*) are located in gene arrays proximal to subtelomeric ends and shared by several chromosomes. In particular, 12/28 subtelomeres in the most distal regions share the array *var(+)/bAa(-)/pseudovar(-) *(where (+) and (-) indicate the orientation with respect to the telomere), while 6 out of 28 are characterized by the following arrangement *var(+)/bAa(-)/cBb(-)*. *RIFIN cBb *is often found associate with *var *pseudogenes and *STEVOR *gene in the array *bAa-pseudovar-stevor-cBb*, 20 copies of this are found in several subtelomeres with little variation.

Our analyses reveal that the overall organisation of *RIFIN *repertoires in different genomes is maintained. While any of the 12 possible combinations may potentially be present in a genome only three arrangements of gene sequences are found frequently in all the three examined clones. The most frequent of these arrangements occur in blocks in blocks of genes which are present in several copies in different subtelomeres.

### Secondary structure prediction of RIFIN proteins

The first description of the putative RIFIN structure predicted a signal peptide and two transmembrane helices on the basis of nine RIFIN sequences from chromosomes 2 and 3 [28]. RIFINs of chromosome 2 were analysed by Gardner and colleagues [29], who predicted a highly conserved transmembrane domain at the C-terminus and an N-terminal signal peptide. No central transmembrane helix was identified in this work. On the other hand, analyses of a typical RIFIN sequence [30], or of RIFIN sequence multiple alignment [15], led to prediction of two transmembrane helices in RIFIN proteins. In order to clarify these controversial results we decided to carry out secondary structure predictions on each of the 159 *bona fide *3D7 sequences.

In order to predict presence of signal peptide sequences in RIFINs we submitted the 3D7 RIFIN amino acid sequences to SignalP 3.0 [31] using both Neural Network (NN) and Hidden Markov Model (HMM) predictions. For each sequence the probability value to contain a signal peptide was considered alongside the most probable position for the cleavage site. We considered a HMM probability higher than 50% to be a reliable prediction for a signal peptide. The result of this analysis was that, surprisingly, while the majority of RIF_Bs (*cds_B *products) (36/46 = 82.6%) possess a signal peptide; about 83.2% (94/113) of RIF_As (*cds_A *products) do not.

**Figure 5:**
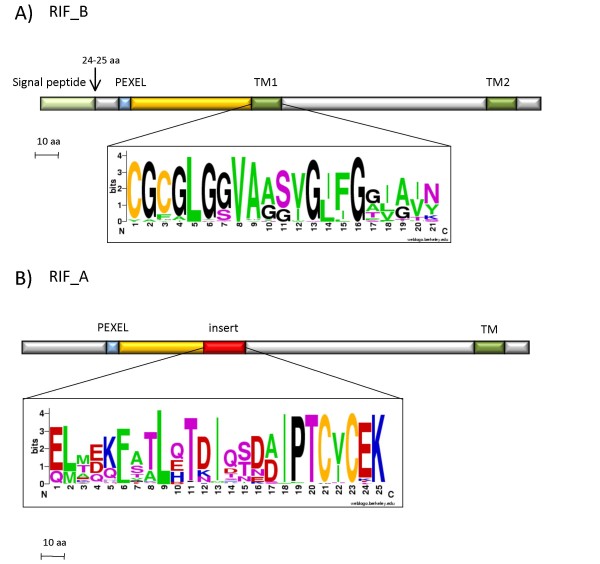
**Results of secondary structure predictions by SignalP and TMHMM**. A) A signal peptide at N-terminus (green), a PEXEL motif (light blue) and two transmembrane helices (TM1 and TM2; dark green) were predicted for RIF_Bs. A sequence logo for TM1 is also shown to highlight a pattern of conserved glycines. B) No signal peptide was predicted for the majority of RIF_As, and only one C-terminal transmembrane helix was found (TM; dark green). Insert (25 aa long) is indicated in red and corresponding sequence logo is also shown. In both RIF_As and RIF_Bs, the region between the PEXEL and the insert is highlighted (yellow). This region is conserved among all members of RIFIN family.

In order to provide a reliable prediction of secondary structures for RIFINs, we submitted RIF_As and RIF_Bs to ConPredII, a consensus based method to predict transmembrane helices (TM) [32]. As expected from previous work, two transmembrane domains were predicted for 45 out of 46 RIF_B (97.8%). The two putative helices are located at positions 121aa ± 6 aa (TM1) and 292aa ± 27aa (TM2), both predicted to be 21 amino acids long. The wider range of positions for TM2 is due to the high variability in the length of the region between the two predicted transmembrane domains (see figure 5, panel A). While TM2 has a typical amino acid composition for a transmembrane helix, TM1 is enriched by glycine residues (sequence logo representation in figure 5). Interestingly, it has been demonstrated that glycines arranged in GxxxG patterns such as those found in RIF_B TM1 promote homodimerisation interactions in transmembrane helices [33]. In fact, the spacing of three residues exposes the glycines on the same side of the helix, allowing very close contact between transmembrane domains and permitting extensive Van der Waals interactions [34].

Surprisingly, a different result was obtained for RIF_As. 99/113 RIF_As have been predicted to contain only one TM, 12/113 two TMs, 1/113 three TMs, while no TM was predicted for PFD1020c. The TM predicted for the majority of RIF_As is at the C-terminus, at positions ranging from 268 aa to 366 aa (figure 5 panel B).

In conclusion, the majority of RIF_As (71.7%) is devoid of a signal peptide, and contains only one TM region, while 35 out of 46 RIF_B RIFINs (76.1%) have a signal peptide and two TM regions. These data were confirmed by analysis of RIF_As and RIF_Bs in HB3 and Dd2 clones. More than 60% of RIF_As (69.7% in HB3 and 67.9% in Dd2) and 50% of RIF_Bs (59.1% in HB3 and 53.1% in Dd2) share the secondary structure organisation of RIFIN found in 3D7.

Our results are consistent with available experimental evidence [17]. In fact, different sub-cellular localisations in intraerythrocytic stages may be explained by structural differences in RIF_As and RIF_Bs. In particular, it is noticeable that the structural organisation of RIF_As resembles that of other antigens like PfEMP1 and PfEMP2, which are exported to the surface of infected erythrocytes, and contain a PEXEL/VTS motif but lack a canonical signal peptide [35].

### *Ab-initio *modeling of RIF_As

As RIFINs have no homologies to other eukaryotic proteins, no information about their putative function can be directly inferred from their amino acid sequences. We therefore constructed a 3D-model for RIF_A proteins to gain insights into their structural/functional features. RIF_A family members were selected for the analysis for their potential involvement in host-parasite interactions. The rationale of the analysis was based on the assumption that all members of the sub-family share a common structure, and took advantage of the large number of RIF_A amino acid sequences to obtain and to quantitatively compare several *ab-initio *3D structure predictions.

The first step of the procedure was the selection of a non redundant set of sequences, which represented the largest possible range of identity percentages between RIF_A members. To this aim, all 113 RIF_A sequences were first aligned pairwise using the Needleman-Wunsch algorithm, and only those sequences which shared a percentage of identity lower than 60% were considered. This reduced the dataset to 53 proteins. In all cases the protein region subject to the analysis was that from the putative site of PEXEL cleavage [36] to the C-terminal TM domain.

**Figure 6:**
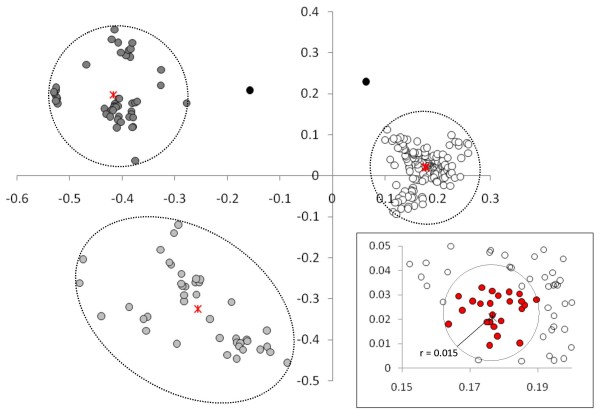
**Multidimensional scaling (MDS) results for 3D-models obtained by I-TASSER for RIF_As (subset of 53 non-redundant sequences)**. The 265 predicted structures were compared by TM-ALIGN, distances were derived (d = 1-TM_score_) and used as input for MDS. Centroids of clusters are shown as red cross. In inset the region around the centroid of the main cluster is reported enlarged. 3D-models within a radius of 0.015 are in red.

The *ab initio*/threading-assembly I-TASSER predictor [19,20] was used to construct 3D-models for the 53 selected sequences. The five top models for the every target sequence were considered and thus 265 models were used in further analyses. To investigate whether a common fold could be identified among the 265 RIF_A models, we first carried out a pairwise comparison over the whole set. All models were structurally aligned pairwise using TM-align [21] and a TM_score _was used to derive a distance (D = 1-TM_score_). MDS was used to visualise relationships between models on a 2D plane as shown in figure 6. We observed that the majority of structural models (177/265) are grouped in a single cluster while other two minor clusters contain the rest of the models (47/265 and 41/265). Since at least one structure for each of the 53 submitted sequences is in the main cluster, we deduced that a common fold has been effectively predicted for members of RIF_A sub-family.

In order to select the most representative models, we considered those within a radius of 0.015 from the centroid of the cluster 1 (see inset in figure 6) and then submitted them to the PROSA [37] and PROCHECK [38] web servers to assess their quality (see Additional file 2). The scores and parameters provided by these two methods along with the scores calculated by I-TASSER were considered and used to establish the best models for RIF_A sub-family. According to this PFF0015c_3 and PFL2660w_5 were thus considered as the most representative models for RIF_As.

**Figure 7:**
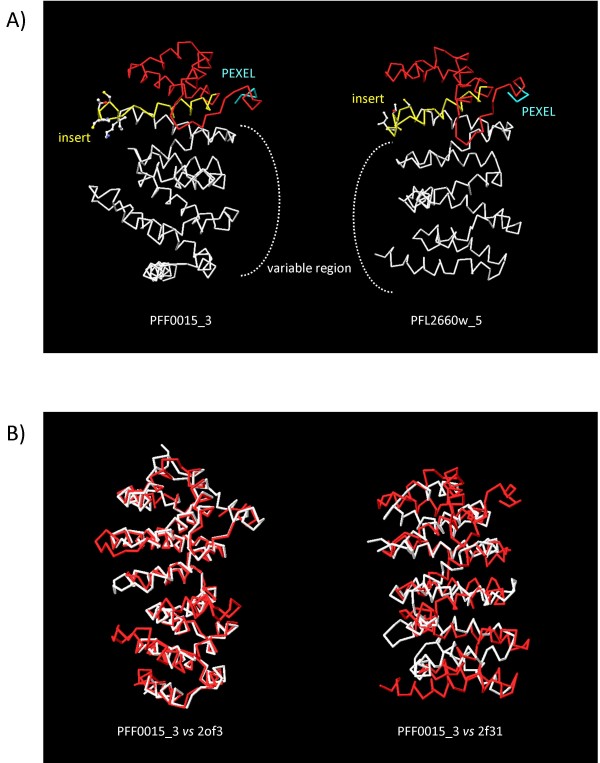
**A) The two most representative 3D-models for RIF_As (PFF0015_c3 and PFL2660w_5) are shown as C-alpha traces in panel A**. PEXEL motif is shown in light blue. Domain corresponding to the conserved region in RIFIN family is indicated in red. Residues conserved in the insertion specific for RIF_As (yellow) are in CPK representation. B) Results of fold matching by SSM, superimpositions between PFF0015c_3 (red) *vs*. 2of3 (white) and PFL2660w_5 (red) *vs*. 2f31 (white) are shown.

Inspection of these two models (predicted structures are reported in figure 7, panel A) revealed that these are alpha-helical structures. The region from the PEXEL/VTS (in light blue in figure 7, panel A) to the RIF_A insert (in yellow), which is well conserved across the RIFIN family, is folded into three helices of about 25 aa joined by two loops (in red). Amino acids in the RIF_A insertion which are highly conserved (see also logo sequence in panel B of figure 5) are shown. We observed that these residues in both models form a loop between two alpha-helices. The sequence from the insert to the TM domain (200 aa long), is highly variable among members of the sub-family (see Additional file 3), in spite of this we observed that this region is in both models arranged in pairs of almost parallel helices of about 15-20 aa joined by short loops. In addition, inspection of other models revealed that this feature is strongly conserved among members of the main cluster (data not shown).

We submitted the models PFF0015c_3 and PFL2660w_5 to the web server ProFunc ([36] to gain insights into the possible role of RIF_As. ProFunc uses a series of methods based on the 3D-structure (fold matching, residue conservation, surface cleft analysis, and functional 3D templates) to help identify possible functions of a protein. Results obtained by fold matching showed that structures predicted for PFF0015c_3 and PFL2660w_5 are folded similarly to two structural domains in PDB database (2of3 and 2f31 respectively, see Table 1). In figure 7 panel B we show the superimposition between RIF_A models (red) and matched structure (white). These structures are both characterised by an "Armadillo-like" fold [40] which comprises two curved layers of alpha helices arranged in a regular right-handed superhelix. These superhelical structures present an extensive solvent-accessible surface that is well suited to binding large substrates such as proteins and nucleic acids.

**Table 1:** Secondary Structure Matching (SSM) parameters

**Query**	**PDB code**	**RMSD (Å)**	**Q-score**	**Z-score**
PFF0015c_3	2of3	1.07	0.657	10

PFL2660w_5	2f31	3.54	0.190	1.7

Root mean square deviation (RMSD) is calculated between the C-alpha atoms of the matched residues in the best 3D superimposition of the query and target structures. The percentage of identity (%ID) is the number of identical amino acids in the structural alignment (%ID is not given in table). Q-score is the ratio between RMSD and the number of aligned amino acids and is considered as a measure of quality of alignment (for identical structure Q = 1). Z-score provides the statistical significance of a fold match in terms of standard Gaussian statistics, the higher Z-score; the higher is the significance of the match

## Discussion

The increasing amount of data from genome-wide experiments (genome sequencing, transcriptomic, proteomic, and interactomic data) with the parallel development of novel bioinformatics tools led to a remarkable improvement of our knowledge about the biology of malaria parasites. In particular, "in silico" approaches allow the annotation of previously uncharacterised proteins [41,42], the identification of possible transcription start sites [43] as well as candidates for transcription factor binding sites [44-46]. In addition, efforts have been devoted in structural genomics experiments  with the aim to identify novel targets for drug and/or vaccine development.

In this framework we carried out a "in silico" study on RIFINs, the most abundant multigene family in *P. falciparum *genome whose products are potentially involved in host-parasite interactions. In 3D7 the family numbers about 159 members and it varies in other clones. In fact, the subtelomeric regions where these genes are located are subject to frequent recombination events leading to a high variability between genomes. Recently, additional sequencing data have become available for other *P. falciparum *clones such as HB3 and Dd2 differing in geographical origin and phenotypic characters.

In this work we exploited genome sequence data to carry out a comparative analysis on RIFIN repertoires between 3D7, HB3 and Dd2. Comparisons were carried out by means of MDS on coding as well as upstream and downstream regions. We found that corresponding sequences have a clear cluster structure which is maintained in the three examined clones. Furthermore, when we compared the observed occurrences of all 12 possible combinations *ups-cds-dwn *with those expected on the basis of a simple probabilistic model, we found very similar distributions of subsets of such combinations in all the three genomes. In addition, despite the high recombination rate of subtelomeric regions and hence the high expected sequence variability, the majority of genes is conserved between clones (i.e. it is possible to identify pairs or triples of orthologs) as well as their cluster organisation. Our results confirm recent studies by Wang et al. [47] which recognised diverse groups of sequences and demonstrated that subsets of genes are highly conserved across genomes. In addition, in the case of upstream and coding sequences we identified several outliers, while none or few were found for downstream sequences. Since outliers may be interpreted as novel sequence variants which are generated by genetic drift, their high numbers in 5' upstream and coding sequences, compared to the low number in 3' downstream sequences indicate that the portions of genes diverge differently.

All these data may be interpreted as a consequence of a balance between drift and homogenisation mechanisms acting on these subtelomeric genes. On one hand, this balance guarantees the emergence of novel gene variants, while on the other; it preserves the functionality of the diverse parts of genes (included gene products) and the overall organisation of the entire repertoire.

In the second part of the work we examined the amino acid sequences of RIFINs in the 3D7 clone. It is already known [14,15] that RIFINs can be grouped into two subfamilies: RIF_As and RIF_Bs. The main difference between members is due to an insert sequence of 25 aa which is present only in RIF_As. In the last few years it has been proposed that despite these differences, RIF_As and RIF_Bs share a similar architectural organisation: a signal peptide at N-terminus; a PEXEL motif; two transmembrane domains, the second of which is C-terminally located. In this work, we carried out a detailed analysis of all the 159 *bona fide *amino acid sequences of RIFINs in 3D7 and submitted every sequence to signal peptide and transmembrane domain predictors [31,32]. Interestingly, while RIF_Bs structural organisation corresponds to that proposed previously, for the majority of RIF_As no signal peptide and only one transmembrane domain at C-terminus were predicted, and hence we proposed different structural architectures for members of the two-sub-families. This is in accordance with Petter et al. [17] which demonstrated that RIF_As and RIF_Bs have different sub-cellular localisations. During the intraerythrocytic stages of life cycle of *P. falciparum*, only RIF_As are exported outside the parasite cell, while RIF_Bs remain confined within the PV. In addition, our results suggest that a canonical signal peptide is necessary to target RIFINs to the PVM or to other sub-cellular compartments, whereas alternative signals are required for translocation outside the parasite cell as demonstrated at least by the other two antigenic proteins PfEMP1 and PfEMP2 [35].

Since RIF_As are those likely to be involved in host-parasite protein interactions, we constructed a 3D-model for the portion of the protein between the putative PEXEL cleavage site [36] and the N-terminus of the C-terminal TM. To do this we applied an *ab initio *procedure starting from the output of the I-TASSER algorithm [20,21]. Taking advantage from the high number of RIFIN sequences, we developed a strategy to determine the most reliable 3D-model for RIF_As using a subset of 53 non-redundant RIF_A sequences. Five 3D models were constructed by I-TASSER for each of the 53 sequences. When all 265 models were then compared using MDS, we observed that they clustered into three groups, the main group of which contains 177/265 predicted structures with at least one structure predicted for each RIF_A family member.

In order to establish the most reliable structures for RIF_As, we selected 24 models within a radius of 0.015 from the centroid of the main cluster in the MDS plane. These structures were submitted to standard methods (PROCHECK, PROSA) for assessing the model quality. The best models PFF0015c_3 and PFL2660w_5 were chosen as representatives and then analysed to try to gain insights into RIF_As function. We found that both structures strongly resemble the "Armadillo-like" fold [40]. This fold is characterised by an arrangement of alpha-helices which form a wide cleft with an extensive solvent-accessible surface and is particularly suited to binding large substrates. In fact, this fold has been found in a wide range of proteins involved in very diverse cellular processes in which protein-protein interactions play an essential role. In particular, the structure matched by PFF0015c_3 is the Tog domain from *C. elegans *gene Zyg9 (2of3). These domains are found in members of the XMAP215/Dis1 family of microtubule-associated proteins (MAPs) which are essential for microtubule growth and probably bind tubulin dimers and promote microtubule polymerization [48]. The structure matched by PFFL2660w_5 (2f31) is the N-terminal regulatory domain of Diaphanous-related formins (DRFs) which regulate the nucleation and polymerisation of unbranched actin filaments [49].

To our knowledge these data represent the first attempt to propose a structural model for the RIF_A proteins of *P. falciparum *based on an *ab-initio *approach implemented on the entire gene family, integrated by an MDS-based assessment of the similarities amongst the obtained 3D predictions. Importantly, these results predict a protein fold which suggests that RIF_As may participate in protein-protein interactions. Further work will be needed to establish the cell compartments where this domain is accessible for such interactions, and to identify the host and/or parasite partners involved.

## Conclusion

In conclusion we found that the overall organisation of *RIFIN *repertoires is maintained in three different clones of *P. falciparum *and that the nucleotide sequences of these genes evolve differently. Furthermore, secondary structure predictions on *RIFIN *products showed that members of the two sub-families RIF_As and RIF_Bs have different architectures. Finally, we proposed a possible role for RIF_As on the basis of *ab initio *3D models.

## Methods

### Data source

3D7 sequences were downloaded by PlasmoDB , while Dd2 and HB3 sequences were obtained by Broad Institute of MIT and Harvard .

### Multidimensional scaling

MDS was carried out on three sets of nucleotide sequence data: 5' sequences (1 kb upstream the first ATG codon), coding sequences and 3' sequences (1 kb the stop codon). Sequences in each group were compared to each other by Needlman-Wunsch pairwise alignments from which the percentages of identity were derived. Distances between sequences were then calculated as d = (100-%ID)/100 and collected into matrices. These matrices were used as input for MDS. Starting from relative distances this statistical method provides a mapping of the given objects (in our case sequences) onto a two-dimensional space and hence allows easy investigation of relationships among them. For each MDS a parameter was calculated that is the stress value (*s*). This parameter ranges from 0 and 1, and measures the degree of correspondence between the distances among objects in the plane and those in the original matrix. In all our analyses we obtained *s *values lower than 0.2 meaning that distances in the plane are a reliable representation of distances between sequences. 3D7 was used as a reference; homologs in the other two clones were identified and then represented on the MDS plane according the cluster organisation established for 3D7.

Possible outliers were determined by calculating every sequence distance from the centroid of cluster whose coordinates are x_c _= Σ_i _x_i_/N, y_c _= Σ_i _x_i_/N; where i = 1,..., N and N is the number of sequences, sequences with distances higher than 0.1 were removed.

### Secondary structure prediction

Each RIFIN protein sequence was submitted to SignalP v3.0 ([31]. SignalP 3.0 uses a combination of neural networks (NN) and hidden Markov models (HMM) and provides prediction of cleavage sites and signal peptide/non-signal peptide. We evaluated both NN and HMM outputs and considered as a reliable prediction those with a HMM probability higher than 0.5. RIFIN sequences were also submitted to ConPredII ([29]. This method predicts the transmembrane topology (i.e., the number of TM segments (TMSs), TMs positions and N-tail location) based on a consensus approach by combining the results of several proposed methods: KKD, TMpred, TopPred II, DAS, TMAP, MEMSAT 1.8, SOSUI, TMHMM 2.0 and HMMTOP 2.0.

### *Ab initio *structure prediction

In order to construct a 3D-model for RIF_As, we used an *ab initio *approach and developed a strategy that takes advantage from the high number of available sequences. Firs of all, we selected a non-redundant set of RIFIN sequences. To do this, a pairwise comparison of *all vs. all *was carried out on 101 RIF_A sequences by Needleman-Wunsch alignment. For every alignment the percentage of identity (%ID) was derived to calculate the distance d = 100-%ID. We considered only sequences sharing a percentage of identity lower than 60%, obtaining 53 proteins (covering a percentage of identity from 32% to 60%).

Sequences from the putative PEXEL cleavage site and the N-terminus of transmembrane domain were submitted to I-TASSER program. First, I-TASSER uses LOMETS [50], a meta-threading approach to identify both homologous and analogous templates (and super-secondary structure segments if global templates are not available) for the query sequence from a non-redundant PDB structure library. In a second step, the continuous fragments excised from the consensus threading templates and the super-secondary structures are assembled into full-length models with the threading-unaligned regions constructed from *ab initio *simulations. For each target sequence, 12,000-30,000 structure decoys are generated by the Monte Carlo assembly simulations. In the third step, the structures decoys are clustered by SPICKER [51], and the centroid of each cluster is determined to identify the most representative models. These undergo to a refinement process by further iterative assembly simulations based on the SPICKER cluster centroids. Finally, according to the structural density of the SPICKER clusters and the I-TASSER force field, the five top models for the target sequence were selected; thus in our case 53 × 5 = 265 models were generated. Although I-TASSER starts from the threading templates, the fragment reassembly procedure often generates correct folds even in the cases where there is no correct template structures identified [19].

The most reliable 3D-models were considered as those in a region immediately around the centroid of the main cluster. A region with a radius equal to 0.015 was defined on the MDS plane and the 24 3D-models in it were considered for further analyses. Assessment of 3D-models was carried out by PROCHECK ([38]) and PROSA ([37] at Profunc web server ). PROSA program performs an energy calculation to map the given structure on a distribution based on all structures in PDB data base, while PROCHECK checks the stereochemical quality of a protein structure.

## Abbreviations

MDS: multidimensional scaling; VSA: variant surface antigens; PV: parasitophorous vacuole; NN: neural network; HMM: hidden markov models; TM: transmembrane helix; SSM: secondary structure matching.

## Authors' contributions

EB and KB performed comparative analyses on RIFIN repertoires by MDS. EB analysed results by I-TASSER, contributed to 3D-model construction and contributed to the manuscript. SM and YZ carried out the *ab initio *3D-model prediction. PA, FS and EP participated in the design of the study. EP conceived the study and wrote the most part of the manuscript. All the authors have read and approved the final manuscript.

## Supplementary Material

Additional file 1 **RIFINs in 3D7, HB3 and Dd2**. three lists are reported of selected RIFINs in the three P. falciparum clones 3D7, HB3 and Dd2. Click here for file

Additional file 2 **Assessment of RIF_A 3D-models**. the data provide results of PROSA and PROCHECK programs on the subset of 24 RIF_A 3D-models Click here for file

Additional file 3 **Alignments of RIF_As and RIF_Bs**. Two Alignments of representative members of RIF_As and RIF_Bs are shown. Click here for file
