# Supplementary material for: Revisiting the Plasmodium falciparum RIFIN family: from comparative genomics to 3D-model prediction
Source: BMC Genomics. 2009 Sep 21;10:445. doi: 10.1186/1471-2164-10-445 (PMC2756283; doi:10.1186/1471-2164-10-445)
Supplement: Additional file 3 — Alignments of RIF_As and RIF_Bs. Two Alignments of representative members of RIF_As and RIF_Bs are shown. [file 1471-2164-10-445-S3.doc]

**R.L.E YD.D RQ..QR..EY.ER....R.R.RE.....IQ.II.KDK..K.L.EK.E C..CGCGL.GGV....G..G.......K..A...A**

Signal peptide

PEXEL

conserved charged domain

TM1

1. RIF_B

PFI0050c MKLQYYKILLFSISLSIFFSSSYA-----HNKNKRYITPQ-TRTTTSRVLGECDLYMPNYDKDADMKSVKENFDRQTSQRFDEYEERMQEKRKKRKEQCDKDIQEIILKDKIEKSLEEKVEKCCLICGCGLGGGVTPFIGLFGGLAVNEMKKVAAVAATE----------------VGIKEAIK----ELGKIFELEAGSNMQWTKMIN

PFE1630w MKVHCYNILLFSLLLHRLLLSSSKVNIEMNHHNTPHIKNR-EPTKSYRSLCECELYTYIYDDDPDMKEIMNDFDRQTSQRFEEYNERVNKNRQKCKEQCDRDIQNIILKDKVEKSLSEKVEKGCLRCGCGL-GGVAAGVGIFGALGTYGWKVAATATAIEFAT------------QEGIKAGIQAA-IEQIKITVFNSLLNVEWLNFIN

PFA0710c MKLHYSKILLFLIPLNTLVTSSSYV----HNKNKPYITPRHTPICTSRVLSECDKQSSIYDNDADMKSVKENFERQTTQRLREYDERMKDKRKKRKEERNKNIQKIIDKDKMEKSLAEKVEIGCLRCGCGL-GGVAASVGLFGGLGTYGWKSAAIATATNAAM--------AEATAKGAEAGVKTLIDKLKEAFPIPNLFDPSFEKAIT

PFI0025c MKLNYTKILLFFFPLNILVTSYHA-----YNKNKPYLKTRHTPRYTSRVLSECDIRSSIYDNDAEMKSVKGTFDRQTSQRFEEYQERIKDKRQKRKEERDKNIQEIIEKDKMDKSLAEKVETGCLKCGCGL-GGVAAGVGIIGGIAINIWKPTALDAAIEAAI--------AKSAAKGATAFKSAVIEGIEKEFGVSILRVHRFESFID

PFA0030c MKLHYTKLLLFFFTLNILLTSYHA-----HNKNKPSITPHHTRSTTSRLLSEYDTESSIYDSDDEIDSVKEIFERQASQRLREYDERLQEKRQKRKEQRDKNIQKIIHKDKMEKNLAEKIEKGCLRCGCGL-GSVAGSIGLFGAVAINIWKPAALDAAITAALNANAVKIAAAANAAGEVIGKA-LVIAELQKMGISTLKGQTLKSFFR

TM2

PFI0050c AGNYSQKMSLVEIVTI-LKNKCEEDEALAGP------------------LFCKASKAATESGEVFEFSGNISRMAADAADAARKAANE---------------KLAEMTSVGTICSNPVVISAIVVVIIAVILLIIYLILRYRRKKKMKKKEQYTKLLNQ

PFE1630w ASNYNSIAGLVEAVKA-----------------------------------AVVSTERTSELSSNTMDR-VRNALSEAENWFSPAVREGTQTTASTITTVQRTQLVDVTATSTYSYMAIAYSVIAILIIVLVMIIIYLILRYRRKKKMNKKAQYTKLLNQ

PFA0710c TETYCNEALISGAVKQKYELLCAKGSS----------------CEN-YSLFSHYRSGGEAEGARALVEG-VKKIVGEA---MGKASEETLKATESQMASLKSGKLAEISETSYSSYRAIGYSVLAILIIVLVMIIIYLVLRYRRKKKK------------

PFI0025c VENYMKVSVISEKVYSHYNTTCIPSGSVHLPS----------VSDPICTLVSKKLPHASQGSSRTVIESTVKTVVSEAEVSAGMAVKK------ATDEAIQR-SIGVVDAKYAICQTAIIASVVAILIIVLVMIIIYLVLRYRRKKKMNKKLQYTKLLNQ

PFA0030c TISYKNVSSITKAVYGQHYEICVYDPSRNLLSSFGDVNRHIGICNSVWKQISAVSQRGQYISHEEVIKRTVETMMSEAEVSAKAAAKT---AEAANKLAIEEAQEQVMEATIYNWYTTIGYTILAILIIVLIMIIIYLILRYRRKKKMKKKAQYTKLLNE

1. RIF_A

PEXEL

conserved charged domain

insert

**R.L.ECELY ..............T.QRF..Y..R....R..CK..CDKEI.KIILKD..EK TL.T.I....IP.C.C..S...K.EK.CL.C....G**

PFC0035w MRITIKMKVHYINILLLALSLNIL-----ANTHQKPSSTPRHI------QTTRLLCECELYM-HNYDNDPEMKRVMQQFHDRTTQRFQEYDERLQEKRQVCKDTCDKEIEKIILKDKIEKELNEKFATLQTDIHSDAIPTCICKTSMADKVEKTCLKCTQNLGGIVA---PSSGVLAGIAEGALYAWKPNALQTAIEAALKAAADDILVGGIEAGK-KV

PFD0040c ------MKIHCINILLFSVPLNIL-----VNTHKKSSIRRLHT------QTTRSLCECELYSPANYDSDPQMKEVMENFIKQTQQRFHDYDDRMKEKRKQCKERCDKEIQKIILKDKLEKELMDKFATLHTDIQSDSIPTCVCEKSLADKTEKFCLNCGKTMG-GVA---PGWGLVGG---LWYATWSQYVTKTAIQKGIEAGVKWGIQE-------LK

PF14_0769 ------MKVHYINILLFALPLDIL-----EHNKNEPHTTPNHT------QTTRSLCECELYSPANNDNDPEMKRVMQQFEDRTSQRFHEYDERMVEKRMQCKDKCDKEIQKIILKDKLEKQMVEQFSTLQTDIQSDAIPTCVCEKSIEDKVEKGCLRCGSILG-AAM---PELGSVGGSLLYALNTWKPAAIIAAKEAALAEATDLATQAGIDTVVAQL

PFF0025w ------MKIHYTNILLFALPLNIL-----VNTHKKPHTTARHTQK---IPTTRSLSECELYAPVNYYSDPQMKEVMDNFNKQTQQRFHEYDERMKTTRQKCKDKCDKEIQKIILKDKLEKELMDKFATLDTDIQSDAIPSCVCEKSIAEKAEKGCLRCGYGLG-SVA---PMIGLTGS---VAVNVWKTAELAAAMELAKQAGAAAGIKAGHLAGT-KV

PF10_0396 ------MKFNYTNIILFSLSLNILLLSSRVYNKRNHKSIILHTSNENPIKTHRSLCECELYSPTNYDSDPEMKRVMQQFHDRTTQRFHEYDERMKTTRQECKEQCDKEIQKIILKDRLEKELMDKFATLHTDIQSDAIPTCVCEKSLADKTEKFCLNCGVQLGGGVLQASGLLGGIGQ---LGLDAWKAAALVTAKELAEKAGAAAGLKAGDIHGM-KI

PFB0040c ------MKDHYINILLFALPLNIL-----VYNQRSYYITPRHT------ETNRSLCECELYSPTNYDSDPEMKRVMQQFEDRTSQRFHEYEERMQSKRMQCKEQCDKEIQKIILKDKLEKELMDKFDTLHTDIQSDAIPTCVCEKSLADKVEKGCLRCGYGLG-TVA---PTVGLIGA---IAVNEWTKAATAAATQKGIEAGI-------------NV

PF10_0400 ------MKFSYFNILLFSIPLNIL-----INDQRNHKSTTHHTLK---IPITRLLCECELYSPDNYDNDAEMKRVMQQFEDRTSQRFHEYDERMQSKRMQCKDRCDKEIQKIILKDKIEKELSQHLSTLETNIDTNDIPTCVCEKSLADKVEKGCLRCGYGLG-TVA---PTVGLIGA---VAVNELKKAAMAIAIKDAIAEGLVAGETARIQASI-KA

TM2

PFC0035w VIGGLDAL-EIEKLGIGSWEPYFTEGYCINVKSLASIIYEKRQTLCG--------ATKSTLDKATCEQIGISIGTMQHDGTYGAPGTTPIETVLNGIVEGTKEAADVAAEAARESATNAIKVQETRLLEAGFNSSISSINASIIAIVVIILIMVIIYFILRYRRKKKLKKKLQYIKLLEE

PFD0040c VFHSLYRLIEVSQ-----IQSFINPANYAEKTTYFSFVKSVNSTKC---------VGKAVNTEPFCN-------------FVSLNGESALSDRAAVIAKDAAYMAEVAKEGVLKEGAS------------VTSSLTTGITASLIAIVVIVLIMIIIYLVLRYRRKKKMKKKLQYIKLLEE

PF14_0769 KIEGLLASFTVKQ-RLVDLSSIVTSSTYNNGAILHKSAMELASSYCH--------FEGTQSTPPFCS---------------------TIKYGQTTNFVRYAKAGSAAFKTEFASKSATLTKAKVGAVEATYGGYHISIISSIVAIVVIVLIMVIIYLILRYRRKKKMKKKLQYIKLLEE

PFF0025w VIDQLHTL-GIYFVGGKPLESIIHVTNYMNVSVIYDKVYSHYTTLCTPRFVIDRPVGDFIFSGPVCN----LVQPNHQGIWVKSSAQAIIKKKVEEAVAEGTQAADVVAKNTADEVTKAAIKTSTEAIDAATTTYYTPIIASIVAIVLIVLIMVIIYKILRYRRKRKMKKKLQYIKLLEE

PF10_0396 VIEGLKAL-KVDTLKSGIFNSFVNNSHYTEVTGLAIAIDTEMNEVC---------SATYIGIHPICV-VREKLGVIPKAGGTMVKQKDAITNVLKQALEKATQSAEALSETTAEDVAAKLTAQKTGAINTIFMSNQTAIIASIVAIVVIVLIMVIIYLILRYRRKKKMKKKLQYIKLLEE

PFB0040c VIDTLKRLFNIEVVTDLKWKTLITAQNYTDKILVGDVIRKLGNTLC---------GGSEDTAGGFCL---------------FTVKANTLPQAINGHVTKAISEGTAEVVKVTE--------AEMGKVTTSAGAYSTGIIVSVVAIVVIVLIMIIIYLILRYRRKRKMTKKMQFMKLLNE

PF10_0400 VILGIKSKFRIDTLGGEVLESIITAQKYDDVSLISESIYMQYQSTCLPQYV----GHGADLSKPICH-TVYTLDFVQGKVHVPGSLQGSIKKALEKIVAEAKSNAVSETANVTTRQTAVFESRNIAAVDATYASYQTAIVASVVAILVIVLVMLIIYLILRYRRKKKMKKKLQYIKLLKE

Alignment of two subsets of RIF_B and RIF_A sequences. Sequences were chosen to cover a wide range of percentage of identity.

1. Residues conserved among members of each sub-family are highlighted: hydrophobic aa in green; negatively charged aa in red; positively charged in dark blue; polar aa in light blue, cysteines in yellow; glycine in pink.
2. TM1 in RIF_Bs and the corresponding region in RIF_As are boxed in red
